# Data Resource Profile: The General Practice Database—Amsterdam, North Holland, Almere (GPD-ANHA)

**DOI:** 10.1093/ije/dyag175

**Published:** 2026-09-05

**Authors:** Jos P Kanning, Habibollah Pirnejad, Awa A Gassama, Coralie R Arends, Johanna H K Joosten, Natasja D Vijfschagt, Ralf E Harskamp, Otto R Maarsingh, Mirjam I Geerlings, Monika Hollander

**Affiliations:** Department of General Practice, Amsterdam UMC, Vrije Universiteit Amsterdam, Amsterdam, The Netherlands; Methodology and Digital Health, Amsterdam Public Health, Amsterdam, The Netherlands; Department of General Practice, Amsterdam UMC, Vrije Universiteit Amsterdam, Amsterdam, The Netherlands; Aging & Later Life and Personalized Medicine, Amsterdam Public Health, Amsterdam, The Netherlands; Department of General Practice, Amsterdam UMC, Vrije Universiteit Amsterdam, Amsterdam, The Netherlands; Department of General Practice, Amsterdam UMC, Vrije Universiteit Amsterdam, Amsterdam, The Netherlands; Department of General Practice, Amsterdam UMC, Vrije Universiteit Amsterdam, Amsterdam, The Netherlands; Department of General Practice, Amsterdam UMC, Vrije Universiteit Amsterdam, Amsterdam, The Netherlands; Department of General Practice, Amsterdam UMC, University of Amsterdam, Amsterdam, The Netherlands; Cardiomyopathy and Arrhythmia, Amsterdam Cardiovascular Sciences Institute, Amsterdam, The Netherlands; Personalized Medicine, Amsterdam Public Health, Amsterdam, The Netherlands; Department of General Practice, Amsterdam UMC, Vrije Universiteit Amsterdam, Amsterdam, The Netherlands; Aging & Later Life and Personalized Medicine, Amsterdam Public Health, Amsterdam, The Netherlands; Department of General Practice, Amsterdam UMC, University of Amsterdam, Amsterdam, The Netherlands; Neurodegeneration, Amsterdam Neuroscience, Amsterdam, The Netherlands; Department of General Practice, Amsterdam UMC, Vrije Universiteit Amsterdam, Amsterdam, The Netherlands

**Keywords:** primary care, database, dynamic cohort, routine care, general practice

Key Features

- The General Practice Database—Amsterdam, North Holland, Almere (GPD-ANHA) is hosted by the Department of General Practice of the Amsterdam University Medical Center (Amsterdam UMC) and was established in 2024 through the integration of the general-practice databases from the former Academic Medical Center and the VU University Medical Center. Its aim is to support research that improves the quality and delivery of primary care.
- The database is a quarterly-updated dynamic cohort, with routine-care data from ∼1.3 million individuals across >160 general practices in the Amsterdam, North Holland, and Almere areas. As of 1 January 2024, 592 762 individuals were actively registered, with a median follow-up of 7.1 years. Data are available at the individual, consultation, and practice levels, and date back to 1996. Available information includes demographics, diagnoses, prescriptions, referrals, laboratory results, vital signs, and unstructured (free-text) consultation notes.
- All data are pseudonymized and harmonized by using a common data model, enabling linkage across practices and other Dutch general-practice networks and cohort studies.
- To date, the GPD-ANHA has supported >45 peer-reviewed international publications.
- Researchers can apply for data access via the GPD-ANHA website or by contacting onderzoekawh-anha@amsterdamumc.nl. Requests are evaluated on scientific merit, feasibility, and relevance to primary care.

## Data resource basics

In the Netherlands, all residents are required to have health insurance and to be registered with a general practitioner (GP). Dutch GPs operate as independent practitioners, typically in group practices, and are primarily funded through a capitation-based reimbursement system via the mandatory health insurance. As gatekeepers to secondary care, GPs manage ∼90% of all healthcare needs while accounting for only 4%–5% of total healthcare expenditure [1]. Dutch general practice is grounded on four core values: person-centredness, medical generalism, continuity, and collaboration [2]. For GPs to perform and document their tasks when providing patient care, they use electronic health records (EHRs) embedded within General Practice Information Systems. These systems support clinical work, information exchange, and continuity of care, while also enabling GPs to meet their legal responsibility to maintain accurate and comprehensive records over a patient’s life course [3].

Beyond their clinical purpose, EHRs are increasingly recognized as a valuable resource for research [4]. They capture real-world health profiles, providing a robust source of population-level health data and reducing the need for costly clinical trials or new cohort recruitment. Importantly, EHRs enhance the generalizability of research findings by enabling research in populations often under-represented or excluded from clinical studies, such as older adults with multiple comorbidities or individuals from diverse migration backgrounds and socio-economic strata.

The General Practice Database—Amsterdam, North Holland, Almere (GPD-ANHA) is a dynamic cohort in which individuals enter upon registration with GPs who have opted to share their EHR data to improve primary care. The database is centred in the Metropolitan Region Amsterdam and built on established EHR infrastructures from the catchment areas of two academic centres: the Amsterdam Academic Medical Center (AMC; since 1996) and the VU University Medical Center (VUmc; since 2003, Fig. 1).

**Figure 1:**
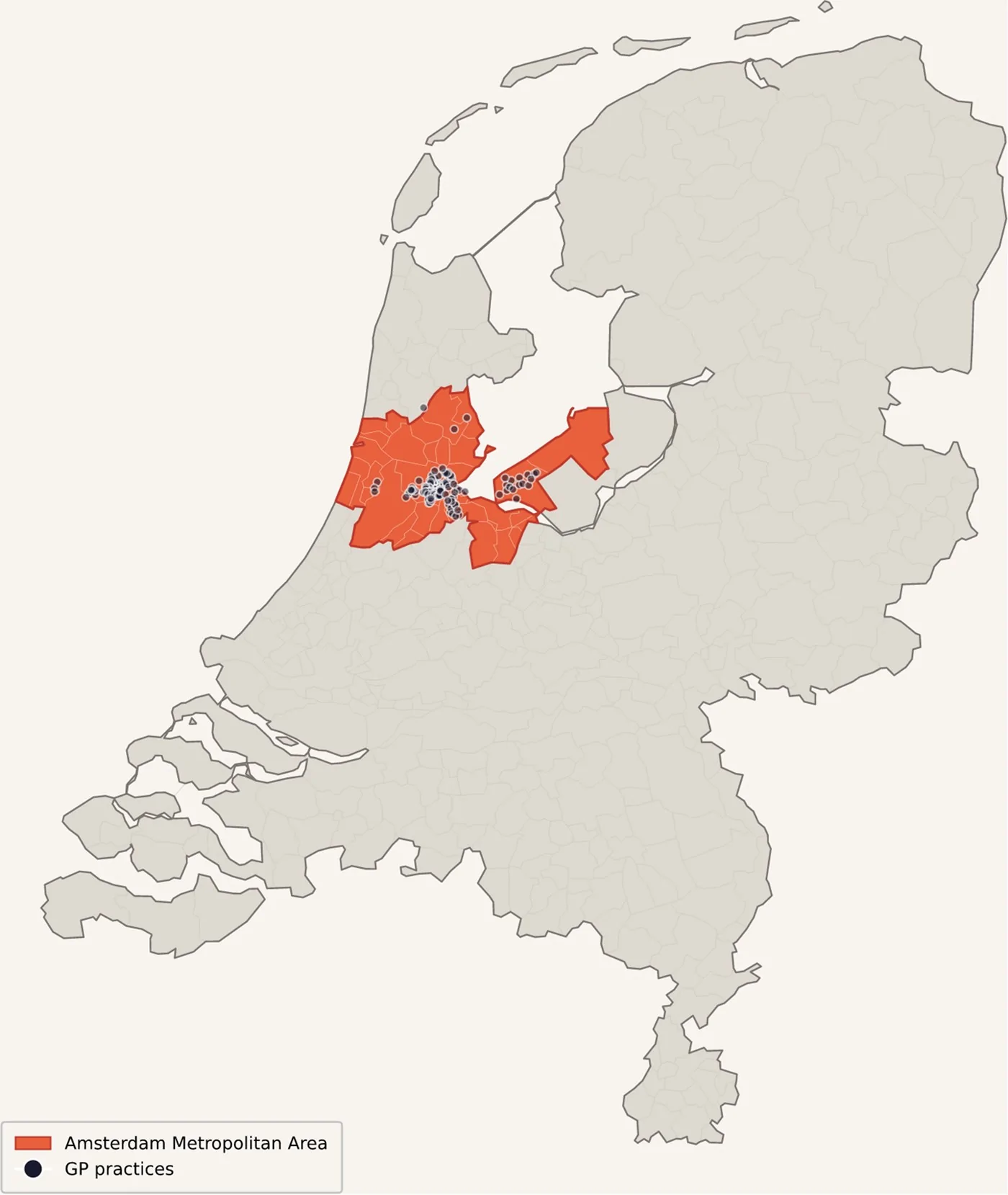
GPD-ANHA coverage within the Netherlands on 1 January 2026. Dots indicate practices affiliated with the GPD-ANHA.

In 2020, the AMC and VUmc general-practice networks were formally integrated within the Academic Network of General Practice Amsterdam UMC (ANHA), aimed at strengthening collaboration among GPs, researchers, and educators to enhance the quality of primary care. This collaboration culminated in 2024 with the integration of both EHR databases into the GPD-ANHA. In 2025, ANHA was renamed AWH-ANHA (Academic General Practice Living Lab—Amsterdam, North Holland, Almere) to reflect its expanded geographic scope (Supplementary Figure 1).

The GPD-ANHA comprises EHR data from >160 general practices centred on the Metropolitan Region Amsterdam, currently covering >1.3 million individuals and expanding rapidly (Supplementary Figure 2). Of these, 592 762 individuals are currently active, defined as being alive and registered at a GPD-ANHA-affiliated general practice as of 1 January 2024 (Table 1).

**Table 1:** Individuals covered in the GPD-ANHA with demographics, risk factors, and disease history on 1 January 2024, based on International Classification of Primary Care coding.

Variable (ICPC)	**Active** ^a^ **(*n* = 592** **762)**	**Inactive (*n* = 725** **694)**
Age [mean (SD)]	39.0 (21.8)	38.0 (22.7)
Age ≥75 years [*n* (%)]	34 693 (5.9)	68 303 (9.4)
Female [*n* (%)]	303 952 (51.3)	386 181 (53.2)
Follow-up (years) [median (IQR)]	10.0 (3.8–19.9)	4.8 (1.9–10.3)
Contacts in past year [mean (SD)]	3.5 (5.2)	2.5 (6.4)
Frequent attenders^b^ [*n* (%)]	47 194 (8.0)	44 998 (6.2)
Smoking status, current [*n* (%)]	29 240 (4.9)	16 508 (2.3)
Smoking status, unknown [*n* (%)]	445 252 (75.1)	658 706 (90.8)
Alcohol abuse (P15, P16) [*n* (%)]	14 829 (2.5)	11 361 (1.6)
Hypertension (K86, K87) [*n* (%)]	72 951 (12.3)	52 110 (7.2)
Heart failure (K77) [*n* (%)]	5669 (1.0)	13 880 (1.9)
Angina pectoris (K74) [*n* (%)]	12 665 (2.1)	12 144 (1.7)
Myocardial infarction (K75) [*n* (%)]	9084 (1.5)	10 499 (1.4)
TIA (K89) [*n* (%)]	6835 (1.2)	7034 (1.0)
Stroke (K90) [*n* (%)]	9431 (1.6)	13 256 (1.8)
Aortic aneurysm (K99.01) [*n* (%)]	2434 (0.4)	2662 (0.4)
Diabetes (T90) [*n* (%)]	35 259 (5.9)	30 319 (4.2)
COPD (R95) [*n* (%)]	11 240 (1.9)	15 087 (2.1)
Infectious diseases (A7, D70, D73, R74, R76, R80, R81, R82, U71) [*n* (%)]	359 189 (60.6)	213 301 (29.4)
Cancer (A79, D74, D75, D76, D77, R84, R85, L71, X75, X76, X77) [*n* (%)]	19 291 (3.3)	26 199 (3.6)
Joint disorder (L17, L20) [*n* (%)]	307 432 (51.9)	139 087 (19.2)
Dementia (P70) [*n* (%)]	2540 (0.4)	10 343 (1.4)
Depression (P76) [*n* (%)]	52 106 (8.8)	32 637 (4.5)

a Active individuals are individuals alive and registered at a GPD-ANHA practice as of 1 January 2024. Conversely, inactive individuals are individuals whose data are contained within the GPD-ANHA and who are no longer registered due to death, emigration, or other forms of deregistration.^b^Defined as having >10 contacts (physical or digital) with the GP in the last year of registration [5]. COPD, chronic obstructive pulmonary disease; ICPC, International Classification of Primary Care; IQR, interquartile range; TIA, transient ischaemic attack.

Follow-up time is defined as the interval between an individual’s registration date at a participating general practice and their deregistration date, which may occur due to transfer to another practice, death, or other reasons. Based on this definition, the median follow-up duration is 7.1 years overall and 10 years among active individuals. Importantly, the EHRs may contain clinical information that predates the formal registration date at a GPD-ANHA practice. As a result, while the follow-up time reflects observation within the GPD-ANHA network, the recorded clinical histories for many patients extend further back in time than their registration dates.

**Figure 2:**
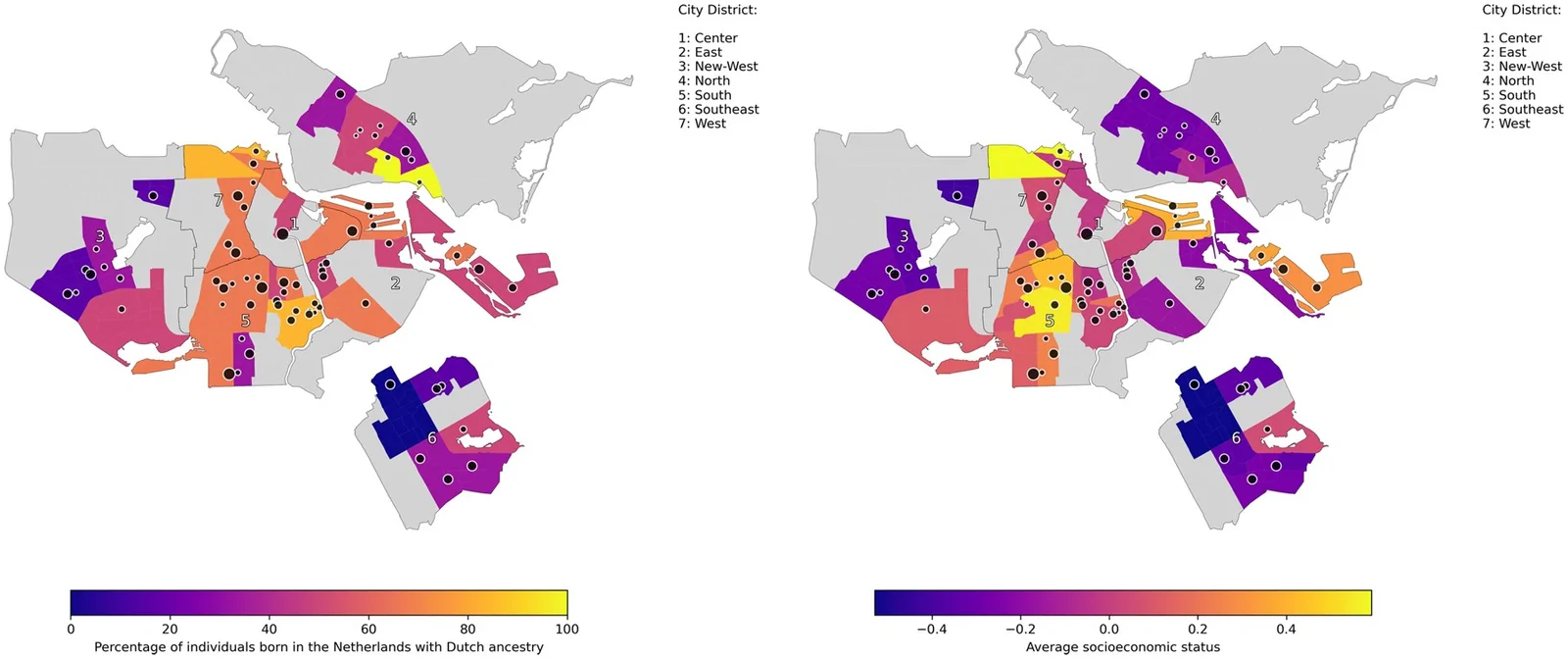
The GPD-ANHA network spans a region characterized by a diverse ethnic and socio-economic population. Each dot represents either an individual practice or an agglomeration of practices located on the same six-digit postal code. The size of the dot is proportional to the number of individuals associated with that practice. Socio-economic status is a composite score calculated by Statistics Netherlands (CBS), consisting of income, education, and employment history [6].

The core content of the EHRs includes demographics (e.g. year of birth, biological sex), consultation records, laboratory results, prescriptions, episodes of care, diagnoses, and free-text notes from GPs. Owing to Amsterdam’s metropolitan character, the database represents a large and diverse population with substantial variation in migration backgrounds, socio-economic status, and financial well-being across districts (Fig. 2 and Supplementary Table 2).

The GPD-ANHA provides a strong foundation for biomedical research and can be linked to other cohorts and registries to address broader research questions.

In accordance with Dutch and European legislation, all GP practices affiliated with the GPD-ANHA inform individuals about the use of routinely collected healthcare data for research and quality improvement. Individuals can opt out at the practice level through an informed opt-out procedure. GP practices newly joining the GPD-ANHA must give individuals sufficient time to be informed about the use of their data for research purposes. Therefore, data extraction for new practices begins only 1 year after registration. Opt-outs are rare, with <0.05% of individuals choosing to do so.

As the GPD-ANHA data are observational, enlisted individuals are not approached individually for participation. Consequently, Medical Ethics Committees in the Netherlands do not consider such research to be subject to the Medical Research Involving Human Subjects Act (WMO; see ethics approval). Nonetheless, researchers must comply with privacy regulations, which is ensured by requiring the signing of both a confidentiality agreement and a data-sharing agreement prior to granting access to the data in a secure environment.

The GPD-ANHA is primarily funded by the Amsterdam UMC and through access fees paid by researchers, with additional support from the Amsterdam Alliance of GPs (AHa). Supplementary funding is occasionally obtained through competitive grants from national research organizations.

## Data collected

EHRs accessible to GPs may contain personally identifiable information (e.g. full names, social security numbers) that is prohibited to be included in the GPD-ANHA. To ensure individual privacy, a trusted third party (ZorgTTP) pseudonymizes the data before extraction into the GPD-ANHA [7]. Personally identifiable information (e.g. names, addresses, social security numbers) is transformed into a unique identifier per practice, preserving privacy while allowing the longitudinal tracking of individuals within the GPD-ANHA. ZorgTTP can additionally link individuals in the GPD-ANHA to other datasets, enabling data augmentation while maintaining patient pseudonymity. Birthdates are truncated to the first day of the month and six-digit postal codes are shortened to the first four digits.

In the Netherlands, each GP practice is free to use its own health informatics system, resulting in variation in EHR structures across practices. To address this variation, another organization (Topicus) harmonizes the data, ensuring that each extraction is delivered in a consistent, structured common data model across all practices and health informatics systems [8]. This common data model is shared with other Dutch academic networks through the broader ‘Intercity’ collaboration, facilitating straightforward linkage when larger patient populations are required. The pseudonymization and harmonization process is illustrated through a fictional example in Supplementary Figure 3.

Data from EHRs are extracted quarterly, with each extraction covering 2 years of prior records. This design creates substantial overlap between successive extractions, allowing changes in individual records to be observed over time. Each extraction also includes metadata on individual registration, deregistration, and death, thereby maintaining a dynamic cohort of active individuals.

An in-house-developed program links successive data extractions to unique individuals, enabling the construction of a longitudinal cohort. Each extraction is reviewed by the GPD-ANHA data managers and compared with previous extractions to verify successful linkage. Data management gives special attention to individuals who have changed GP practices (and therefore received a new identifier) and to practices that have switched to a different EHR (which may also result in reassigned identifiers), ensuring the integrity of longitudinal records.

The database includes all information available to a GP within the EHR, such as diagnoses coded using the International Classification of Primary Care (ICPC) system, medications coded using the Anatomical Therapeutic Chemical (ATC) system, and laboratory and clinical measurements (e.g. blood pressure, weight, blood glucose levels) coded using standardized Dutch College of General Practitioners codes, along with consultation records. However, imaging results, referral letters, and hospital correspondence are not included. A detailed overview of the available data at each level has been reported elsewhere [9].

In addition, the GPD-ANHA contains free-text notes recorded by GPs during consultations. These notes follow the Subjective, Objective, Evaluation, and Plan (SOEP) structure [10], which can be made available to researchers after additional depersonalization through a rule-based algorithm that removes potentially identifiable information (e.g. names, contact details).

Data are collected at the individual, consultation, and practice levels, and can be linked to other data sources. Since 2018, the ANH-VUmc and AHA-AMC databases (later merged into the GPD-ANHA) have participated in the Intercity infrastructure, facilitating straightforward linkage with other regional general-practice networks that share a common data model, including the Nivel Primary Care Database (Nivel-PCD) [11], Academic General Practitioner Network (AHON) [9], Julius General Practitioners’ Network (JGPN) [12], and Research Network Family Medicine Maastricht (RNFM) [13]. Furthermore, linkage to national registries, such as Statistics Netherlands (CBS) and the Netherlands Comprehensive Cancer Organization (IKNL) [14, 15], provides supplementary data on demographics, hospital diagnoses, and cancer incidence. Linkage to cohort studies, such as the Healthy Life in an Urban Setting (HELIUS), adds genetic and phenotypic information for a subset of HELIUS participants who are present in the GPD-ANHA [16].

## Data resource use

The GPD-ANHA serves both clinical and research purposes. Among participating general practices, it supports clinical quality improvement through the benchmarking of performance metrics and providing feedback via audit and feedback sessions (Dutch: ‘Spiegelinformatie’). These sessions are held in person, during which GPs discuss specific topics (e.g. medication prescribing for vertigo, atrial fibrillation, and heart failure) and review how their practice compares to others in the network [17]. Such discussions highlight practice variation and promote more uniform standards of care.

To date, the GPD-ANHA and its earlier components have supported >45 peer-reviewed publications. Research spans pandemic-related care and outcomes, cardiometabolic disease, frailty and continuity of care, evaluation of primary care interventions and registration quality, medication safety, and prediction modelling. Illustrative examples include work on delayed cancer referral during COVID-19 [18], characteristics of heart failure [19], sudden cardiac arrest risk in type 2 diabetes [20], frailty identification [21], continuity of care [22], cost-effectiveness of implementation strategies [23, 24], medication use [25–27], and prediction modelling [28], including approaches using the natural language processing of consultation notes [29–31]. A full list of publications based on the GPD-ANHA and its earlier components is available in the Supplementary material.

## Strengths and weaknesses

One of the key strengths of the GPD-ANHA is its size, in terms of both the large number of individuals and the long follow-up period, as well as its diversity, which surpasses that of several other registries in the Netherlands, especially when linked to the Intercity databases mentioned above. This scale enables research on rare outcomes that require large sample sizes, as well as studies on under-represented populations, including individuals with migration backgrounds, non-native Dutch speakers, and expatriates. As every resident of the Netherlands is legally required to be registered with a GP and opt-out from the GPD-ANHA is rare, groups that are often difficult to recruit into cohort studies are well represented in the database. The inclusion of such populations enhances the generalizability of findings and supports a more inclusive understanding of health trends.

Another strength is the frequency of data updates. Quarterly extractions allow the timely investigation of rapidly evolving public health issues, such as the COVID-19 pandemic, and support longitudinal analyses of trends such as shifts in ICPC coding over time [18]. The database is also readily linkable to other datasets, allowing expansion in both depth and breadth. For example, linkage to specialized cohorts such as HELIUS can add genetic and phenotypic data, while linkage to other primary care databases can extend geographic coverage across the Netherlands. These linkages are facilitated by a common trusted third party shared across networks.

Several limitations should be acknowledged. First, there exists a possibility that GPs who share their data with the GPD-ANHA (and, by extension, their registered patients) systematically differ from those who do not. We believe that this is unlikely: our network spans a wide range of neighbourhoods across the Metropolitan Region Amsterdam, encompassing substantial variation in socio-economic status and migration background, which suggests that the patient populations represented are broadly diverse. Efforts to recruit additional GPs in the coming years aim to further strengthen the representativeness of the network. Second, the GPD-ANHA encompasses a wide variety of health information systems, which are subject to change as practices adopt new platforms. Such transitions can lead to the reshuffling of unique identifiers, potentially complicating longitudinal tracking or causing variable availability to change. However, these effects are mitigated by the harmonization performed by another shared trusted third party and by probabilistic matching algorithms developed in-house. Population mobility in Amsterdam, including high turnover among expatriates and frequent movers, can limit follow-up duration. These individuals can be excluded from analyses when longer-term follow-up is required or included when hard-to-capture populations are being studied.

As with all routine-care datasets, data are collected for clinical rather than scientific purposes. As a result, missing data is a concern. Certain variables, such as diagnoses or laboratory measurements, may be inconsistently recorded or absent altogether, and missingness is often guided by clinical practice and related to the value of the missing variable itself. For example, blood pressure is more likely to be recorded in individuals with suspected or known hypertension. Several strategies can help mitigate these limitations, including robust study designs that explicitly account for missing-data mechanisms, careful selection of the study population to minimize systematic gaps, and data augmentation through record linkage with complementary sources. Additionally, many clinically relevant variables are not captured in standardized formats (e.g. ICPC, ATC) and must be inferred through alternative methods. Free-text clinical notes offer valuable contextual information, but introduce technical and legal challenges; text-processing algorithms, including natural language processing approaches, present a promising avenue for extracting structured information from such unstructured sources.

A third limitation arises from the restricted scope of the GPD-ANHA database: any clinical care occurring outside this network will necessarily be absent from our data, potentially obscuring a complete patient profile. However, the impact of this limitation is mitigated by structural features of the Dutch primary care system. Patients are registered with a specific GP, referrals are required for specialist access, and the health-insurance system discourages visits to outside practices by only reimbursing care provided by the patient’s own registered GP. Care delivered in other settings (e.g. out-of-hours services, hospital environments) is typically reported back to the patient’s own practice, preserving data continuity. Data augmentation through the linkages described above may further reduce this gap. Finally, interpreting data from Dutch general practice requires specific domain knowledge. To address this, the GPD-ANHA is embedded within a broad expertise network that connects practitioners, researchers, and educators. In addition to GPs engaged in both clinical care and research, the GPD-ANHA collaborates closely with technical expertise centres such as the Department of Medical Informatics at Amsterdam UMC. This collaboration has produced research-supporting tools, including dashboards and adjudication systems that facilitate clinical annotation for machine-learning applications.

## Data resource access

Researchers may request to work with GPD-ANHA data by contacting our research coordinator (onderzoekawh-anha@amsterdamumc.nl). All requests undergo internal review and are evaluated on scientific merit, feasibility, and relevance to primary care. Upon approval, researchers will have to sign a non-disclosure agreement and may receive de-identified datasets or perform analyses within a secured research environment. Associated costs are initially based on the amount of work estimated by the GPD-ANHA data managers. Additional costs may be incurred through data processing (e.g. storage, compute power).

Additional information (in Dutch) can be found on our website (Amsterdam UMC, Locatie VUmc—ANHA Informatie voor onderzoekers).

## Ethics approval

The medical ethics committee of Amsterdam UMC has concluded that the protocol does not constitute clinical research with human subjects, as defined in the Medical Research Involving Human Subjects Act (WMO). This declaration for the GPD-ANHA registry is recorded in the medical ethics research register of Amsterdam UMC (no. 2025–0954).

## Supplementary Material

dyag175_Supplementary_Data

## Data Availability

See ‘Data Resource Access’, above.
