# Supplementary material for: Data Resource Profile: The General Practice Database—Amsterdam, North Holland, Almere (GPD-ANHA)
Source: Int J Epidemiol. 2026 Sep 5;55(5):dyag175. doi: 10.1093/ije/dyag175 (PMC13546033; doi:10.1093/ije/dyag175)
Supplement: Supplementary file 1 [file dyag175_supplementary_data.zip › ije-2026-01-0101-File006.docx]

**Supplementary Information**

**Figure S1.** The history of the General Practice Database – Amsterdam, North Holland Almere (GPD-ANHA).

AMC HAG-NET: Huisartsgeneeskundig Netwerk van het Academisch Medisch Centrum Amsterdam [General Practice Network of the Academic Medical Center Amsterdam], ANH-VUmc: Academisch Netwerk Huisartsgeneeskunde – Vrije Universiteit Medisch Centrum [Academic Network of General Practice – VU University Medical Center], AHA-AMC: Academisch Netwerk Huisartsgeneeskunde – Amsterdam Medisch Centrum [Academic Network of General Practice – Amsterdam University Medical Center], AWH-ANHA: Academische Werkplaats Huisartsenzorg - Amsterdam, Noord-Holland, Almere [Academic General Practice Living Lab – Amsterdam, North Holland, Almere], GPD-ANHA: General Practice Database – Amsterdam, North Holland, Almere.

**Figure S2.** Growth of the GPD-ANHA over time.

**Figure S3.** Hypothetical flowchart of pseudonymization and harmonization before data is extracted into the GPD-ANHA.

All data used in this example is fictional and does not refer to real-life individuals. GPD-ANHA: General Practice Database – Amsterdam, NorthHollandAlmere, ICPC: International Classification of Primary Care.

**Table S1:** Definitions of risk factors and disease presented in Tables 1 and 2.

| **Risk factors and disease** | **Definition(s)*** |
| --- | --- |
| Contacts past year | NHG** code in ('consultation', 'out-of-hours consultation’, ‘e-consultation’ ‘telephone consultation’, 'out-of-hours telephone consultation’, ‘consultation visit’, ‘out-of-hours consultation visit’ |
| Frequent attenders | Defined as having more than 10 contacts (physical or digital) with the GP in the last year of registration |
| Smoking (yes/no) | NHGnummer = 1739 and any corresponding (text) result that indicates either an affirmative (e.g. ‘yes’, ‘1’), a non-zero quantity (e.g. ‘yes #4’, ‘smokes X cigarettes a day’, ‘smokes a carton a day’), a text that likely indicates an affirmative (e.g. ‘smk’, ‘smo’), or a willingness to quit (e.g. ‘wants to stop’). |
| Alcohol abuse | ICPC codes P15%, P16% |
| Hypertension | ICPC codes K86%, K87% |
| Heart failure | ICPC codes K77% |
| Angina pectoris | ICPC codes K74% |
| Myocardial infarction | ICPC codes K75% |
| TIA | ICPC codes K89% |
| Stroke | ICPC codes K90% |
| Aortic aneurysm | ICPC codes K99.01% |
| Diabetes | ICPC codes T90% |
| COPD | ICPC codes R95% |
| Infectious diseases | ICPC codes A7%, D70%, D73%, R74%, R76%, R80%, R81%, R82%, U71% |
| Cancer | ICPC codes A79%, D74%, D75%, D76%, D77%, R84%, R85%, L71%, X75%, X76%, X77% |
| Joint disorder | ICPC codes L08%, L10%, L11%, L12%, L15%, L16%, L17%, L20% |
| Dementia | ICPC codes P70% |
| Depression | ICPC codes P76% |

*: % indicates a truncation, such that all ICPC subcodes are used in the definition of the risk factor and/or disease. **: NHG codes are standardized clinical coding systems used in Dutch primary care, primarily used to encode information not encoded in ICPC or ATC codes (e.g. consultation and measurement types). COPD: Chronic obstructive pulmonary disease, ICPC: International Classification of Primary Care, NHG: Nederlands Huisartsen Genootschap [Dutch College of General Practitioners.], TIA: Transient Ischemic Attack, SD: Standard deviation.

**Table S2.** Individuals covered in the GPD-ANHA by region of Amsterdam with demographics, risk factors, and disease history on January 1, 2024 based on International Classification of Primary Care (ICPC) coding.

| **Variable (ICPC)** | **Center**  **(n = 112,232)** | **East**  **(n = 123,821)** | **New West**  **(n = 99,981)** | **North**  **(n = 92,470)** | **South**  **(n = 245,772)** | **Southeast**  **(n = 60,446)** | **West**  **(n = 87,354)** | **Total**  **(822,076)** |
| --- | --- | --- | --- | --- | --- | --- | --- | --- |
| **Age, mean (SD)** | 38.5 (20.0) | 35.2 (20.1) | 38.2 (23.2) | 39.0 (24.0) | 40.1 (23.1) | 35.7 (21.9) | 37.0 (19.6) | 38.1 (22.0) |
| **Age ≥75 years, n (%)** | 5,905 (5.3) | 5,355 (4.3) | 9,393 (9.4) | 9,622 (10.4) | 26,328 (10.7) | 3,367 (5.6) | 4,163 (4.8) | 64,133 (7.8) |
| **Female, n (%)** | 58,366 (52.0) | 65,022 (52.5) | 53,034 (53.0) | 48,339 (52.3) | 133,700 (54.4) | 30,864 (51.1) | 46,623 (53.4) | 435,948 (53.0) |
| **Follow-up in years, median (IQR)** | 6.4 (2.3-13.6) | 5.3 (2.0-11.9) | 6.4 (2.2-14.0) | 6.2 (2.2-14.3) | 4.9 (1.8-11.3) | 5.3 (2.0-11.1) | 5.3 (2.0-11.9) | 6.0 (2.3-13.9) |
| **Contacts past year, mean (SD)** | 2.9 (5.7) | 3.5 (5.9) | 3.8 (6.4) | 3.5 (6.0) | 3.7 (7.3) | 4.2 (7.3) | 3.2 (6.1) | 3.5 (6.5) |
| **Frequent attenders*, n (%)** | 7,831 (7.0) | 9,938 (8.0) | 9,910 (9.9) | 8,324 (9.0) | 23,655 (9.6) | 6,877 (11.4) | 6,821 (7.8) | 73,356 (8.9) |
| **Smoking status, current, n (%)** | 3,110 (2.8) | 3,762 (3.0) | 3,289 (3.3) | 3,212 (3.5) | 7,068 (2.9) | 2,039 (3.4) | 3,131 (3.6) | 25,611 (3.1) |
| **Smoking status, unknown, n (%)** | 97,836 (87.2) | 107,327 (86.7) | 84,348 (84.4) | 77,953 (84.3) | 210,292 (85.6) | 51,562 (85.3) | 75,833 (86.8) | 705,151 (85.8) |
| **Alcohol abuse (P15, P16), n (%)** | 2,522 (2.2) | 2,531 (2.0) | 1,747 (1.7) | 1,846 (2.0) | 4,985 (2.0) | 1,337 (2.2) | 2,028 (2.3) | 16,996 (2.1) |
| **Hypertension (K86, K87), n (%)** | 7,934 (7.1) | 8,768 (7.1) | 10,900 (10.9) | 9,711 (10.5) | 21,311 (8.7) | 6,873 (11.4) | 5,074 (5.8) | 70,571 (8.6) |
| **Heart failure (K77), n (%)** | 959 (0.9) | 1,227 (1.0) | 1,794 (1.8) | 1,704 (1.8) | 4,118 (1.7) | 875 (1.4) | 809 (0.9) | 11,486 (1.4) |
| **Angina pectoris (K74), n (%)** | 1,415 (1.3) | 1,514 (1.2) | 2,624 (2.6) | 2,037 (2.2) | 4,468 (1.8) | 943 (1.6) | 999 (1.1) | 14,000 (1.7) |
| **Myocardial infarction (K75), n (%)** | 1,040 (0.9) | 1,176 (0.9) | 1,770 (1.8) | 1,767 (1.9) | 2,729 (1.1) | 729 (1.2) | 765 (0.9) | 9,976 (1.2) |
| **TIA (K89), n (%)** | 821 (0.7) | 822 (0.7) | 1,070 (1.1) | 939 (1.0) | 2,666 (1.1) | 526 (0.9) | 521 (0.6) | 7,365 (0.9) |
| **Stroke (K90), n (%)** | 1,326 (1.2) | 1,469 (1.2) | 2,059 (2.1) | 1,714 (1.9) | 4,001 (1.6) | 1,243 (2.1) | 1,039 (1.2) | 12,851 (1.6) |
| **Aortic aneurysm (K99.01), n (%)** | 343 (0.3) | 279 (0.2) | 387 (0.4) | 384 (0.4) | 1,082 (0.4) | 163 (0.3) | 209 (0.2) | 2,847 (0.3) |
| **Diabetes (T90), n (%)** | 3,604 (3.2) | 4,840 (3.9) | 6,767 (6.8) | 5,896 (6.4) | 9,218 (3.8) | 3,895 (6.4) | 2,758 (3.2) | 36,978 (4.5) |
| **COPD (R95), n (%)** | 1,713 (1.5) | 1,804 (1.5) | 2,283 (2.3) | 2,416 (2.6) | 4,504 (1.8) | 936 (1.5) | 1,523 (1.7) | 15,179 (1.8) |
| **Infectious diseases (A7, D70, D73, R74,**  **R76, R80, R81, R82, U71), n (%)** | 40,565 (36.1) | 55,349 (44.7) | 42,158 (42.2) | 37,926 (41.0) | 92,970 (37.8) | 26,675 (44.1) | 33,343 (38.2) | 328,986 (40.0) |
| **Cancer (A79, D74, D75, D76,**  **D77, R84, R85, L71, X75, X76, X77), n (%)** | 3,231 (2.9) | 3,050 (2.5) | 3,510 (3.5) | 3,290 (3.6) | 9,068 (3.7) | 1,687 (2.8) | 2,271 (2.6) | 26,107 (3.2) |
| **Joint disorder (L17, L20), n (%)** | 32,359 (28.8) | 42,801 (34.6) | 31,300 (31.3) | 30,469 (33.0) | 70,716 (28.8) | 20,185 (33.4) | 25,857 (29.6) | 253,687 (30.9) |
| **Dementia (P70), n (%)** | 503 (0.4) | 589 (0.5) | 1,377 (1.4) | 1,069 (1.2) | 3,302 (1.3) | 745 (1.2) | 522 (0.6) | 8,107 (1.0) |
| **Depression (P76), n (%)** | 6,172 (5.5) | 7,604 (6.1) | 7,589 (7.6) | 6,027 (6.5) | 13,086 (5.3) | 3,485 (5.8) | 5,359 (6.1) | 49,322 (6.0) |

*: Defined as having more than 10 contacts (physical or digital) with the GP in the last year of registration. COPD: Chronic obstructive pulmonary disease, ICPC: International Classification of Primary Care, IQR: Interquartile Range, TIA: Transient Ischemic Attack, SD: Standard deviation.

**Table S3**: Definitions of geographical regions of Amsterdam based on 4-digit postal codes.

| **Region of Amsterdam** | **4-digit postal codes** |
| --- | --- |
| Center | 1011, 1012, 1013, 1015, 1016, 1017, 1018, 1019 |
| South | 1058, 1059, 1071, 1072, 1073, 1074, 1075, 1076, 1077, 1078, 1079, 1081, 1082, 1083 |
| East | 1019, 1086, 1087, 1091, 1092, 1093, 1094, 1095, 1096, 1097, 1098 |
| New-West | 1043, 1046, 1047, 1057, 1058, 1059, 1061, 1062, 1063, 1064, 1065, 1066, 1067, 1068, 1069 |
| Wests | 1013, 1014, 1051, 1052, 1053, 1054, 1055, 1056, 1057, 1058, 1061, 1071, 1075 |
| North | 1021, 1022, 1023, 1024, 1025, 1026, 1027, 1028, 1031, 1032, 1033, 1034, 1035, 1036, 1037 |
